# Qualification of a Biolayer Interferometry Assay to Support AZD7442 Resistance Monitoring

**DOI:** 10.1128/spectrum.01034-22

**Published:** 2022-08-22

**Authors:** Tyler Brady, Tianhui Zhang, Kevin M. Tuffy, Nantaporn Haskins, Qun Du, Jia Lin, Gilad Kaplan, Steven Novick, Tiffany L. Roe, Kuishu Ren, Kim Rosenthal, Patrick M. McTamney, Michael E. Abram, Katie Streicher, Elizabeth J. Kelly

**Affiliations:** a Translational Medicine, Vaccines and Immune Therapies, BioPharmaceuticals R&D, AstraZeneca, Gaithersburg, Maryland, USA; b Data Sciences and Quantitative Biology, AstraZeneca, Gaithersburg, Maryland, USA; c Biologics Engineering, R&D, AstraZeneca, Gaithersburg, Maryland, USA; d Virology and Vaccine Discovery, Vaccines and Immune Therapies, BioPharmaceuticals R&D, AstraZeneca, Gaithersburg, Maryland, USA; Emory University School of Medicine

**Keywords:** severe acute respiratory syndrome coronavirus 2, coronavirus disease 2019, monoclonal antibody, spike protein, receptor binding domain

## Abstract

AZD7442, a combination of two long-acting monoclonal antibodies (tixagevimab [AZD8895] and cilgavimab [AZD1061]), has been authorized for the prevention and treatment of coronavirus disease 2019 (COVID-19). The rapid emergence of severe acute respiratory syndrome coronavirus 2 (SARS-CoV-2) variants requires methods capable of quickly characterizing resistance to AZD7442. To support AZD7442 resistance monitoring, a biolayer interferometry (BLI) assay was developed to screen the binding of tixagevimab and cilgavimab to SARS-CoV-2 spike proteins to reduce the number of viral variants for neutralization susceptibility verification. Six spike variants were chosen to assess the assay’s performance: four with decreased affinity for tixagevimab (F486S:D614G and F486W:D614G proteins) or cilgavimab (S494L:D614G and K444R:D614G proteins) and two reference proteins (wild-type HexaPro and D614G protein). Equilibrium dissociation constant (*K_D_*) values from each spike protein were used to determine shifts in binding affinity. The assay’s precision, range, linearity, and limits of quantitation were established. Qualification acceptance criteria determined whether the assay was fit for purpose. By bypassing protein purification, the BLI assay provided increased screening throughput. Although limited correlation between pseudotype neutralization and BLI data (50% inhibitory concentration versus *K_D_*) was observed for full immunoglobulins (IgGs), the correlations for antibody fragments (Fabs) were stronger and reflected a better comparison of antibody binding kinetics with neutralization potency. Therefore, despite strong assay performance characteristics, the use of full IgGs limited the screening utility of the assay; however, the Fab approach warrants further exploration as a rapid, high-throughput variant-screening method for future resistance-monitoring programs.

**IMPORTANCE** SARS-CoV-2 variants harbor multiple substitutions in their spike trimers, potentially leading to breakthrough infections and clinical resistance to immune therapies. For this reason, a BLI assay was developed and qualified to evaluate the reliability of screening SARS-CoV-2 spike trimer variants against anti-SARS-CoV-2 monoclonal antibodies (MAbs) tixagevimab and cilgavimab, the components of AZD7442, prior to *in vitro* pseudovirus neutralization susceptibility verification testing. The assay bypasses protein purification with rapid assessment of the binding affinity of each MAb for each recombinant protein, potentially providing an efficient preliminary selection step, thus allowing a reduced testing burden in the more technically complex viral neutralization assays. Despite precise and specific measures, an avidity effect associated with MAb binding to the trimer confounded correlation with neutralization potency, negating the assay’s utility as a surrogate for neutralizing antibody potency. Improved correlation with Fabs suggests that assay optimization could overcome any avidity limitation, warranting further exploration to support future resistance-monitoring programs.

## INTRODUCTION

In December 2019, an outbreak of severe pneumonia was reported in Wuhan, China (1). The highly contagious respiratory illness coronavirus disease 2019 (COVID-19) has resulted in an ongoing pandemic associated with significant morbidity and mortality (2).

The causal agent, severe acute respiratory syndrome coronavirus 2 (SARS-CoV-2) (3), is a positive-sense RNA-enveloped virus coated with spike protein. This trimeric protein binds to the angiotensin-converting enzyme 2 (ACE2) receptor on host cells and facilitates entry through fusion of the host cell and viral membranes (4, 5), and thus, it serves as an attractive target for various therapy candidates. Monoclonal antibodies (MAbs) have emerged as important tools in the armamentarium against COVID-19 disease, with multiple MAbs authorized or approved for the treatment of COVID-19 and one MAb cocktail, AZD7442 (Evusheld), being authorized for the prevention of COVID-19. AZD7442 is a combination of two recombinant long-acting MAbs, tixagevimab (AZD8895) and cilgavimab (AZD1061), in clinical development for prophylaxis of COVID-19 and treatment of mild to moderate COVID-19 (6–10). Tixagevimab and cilgavimab simultaneously bind to distinct, epitopes on the spike protein receptor-binding domain (RBD) to neutralize SARS-CoV-2 (11–13).

The geometric mean titers of binding and neutralizing antibodies are highly correlated with the protection offered by vaccines (14, 15). Tixagevimab and cilgavimab have demonstrated potent SARS-CoV-2 neutralizing activity (13). SARS-CoV-2 replication is inherently error prone, resulting in natural polymorphisms. The emergence of SARS-CoV-2 variants harboring amino acid substitutions in the spike protein may reduce the effectiveness of current vaccines and MAbs under development for prevention of COVID-19, leading to breakthrough infections (16, 17). Current evidence suggests that some viral variants can escape neutralization through diminished MAb binding and that combinations of MAbs targeting distinct epitopes on the SARS-CoV-2 spike protein provide a higher threshold against virus escape from neutralization than individual antibodies (18). Recent data confirmed that the neutralizing potency of AZD7442 is largely unaffected by SARS-CoV-2 variants of concern, including Alpha (B.1.1.7), Beta (B.1.351), Gamma (P.1), and Delta (B.1.617.2) (12, 16, 19–23).

Given the potential for altered relative risk reduction for MAbs utilized in prophylaxis and treatment when decreases in binding and neutralizing potency are observed, there remains an unmet need for robust screening assays to allow targeted confirmatory assessment of the neutralizing activity of MAbs against constantly emerging SARS-CoV-2 variants. One potential approach is a biolayer interferometry (BLI) assay to evaluate how amino acid substitutions in the SARS-CoV-2 spike protein impact AZD7442 binding kinetics. BLI assays are readily scalable and, in avoiding the need for time-consuming protein purification, would allow rapid screening of SARS-CoV-2 spike variants and reduce the number of variants that require phenotypic verification in the more resource-intensive pseudotyped-virus neutralization assay (24). Tixagevimab and cilgavimab each neutralize SARS-CoV-2 by binding to the RBD and blocking ACE2 receptor interactions; thus, a strong correlation between a BLI assay and an *in vitro* neutralization susceptibility assay would confirm the utility of the BLI approach as a screening assay.

Here, we describe the development and qualification of a BLI assay for use as a screening method to determine the phenotypic impact of MAb binding to SARS-CoV-2 spike trimer proteins harboring sequence changes identified in virus variants.

## RESULTS

**FIG 1:**
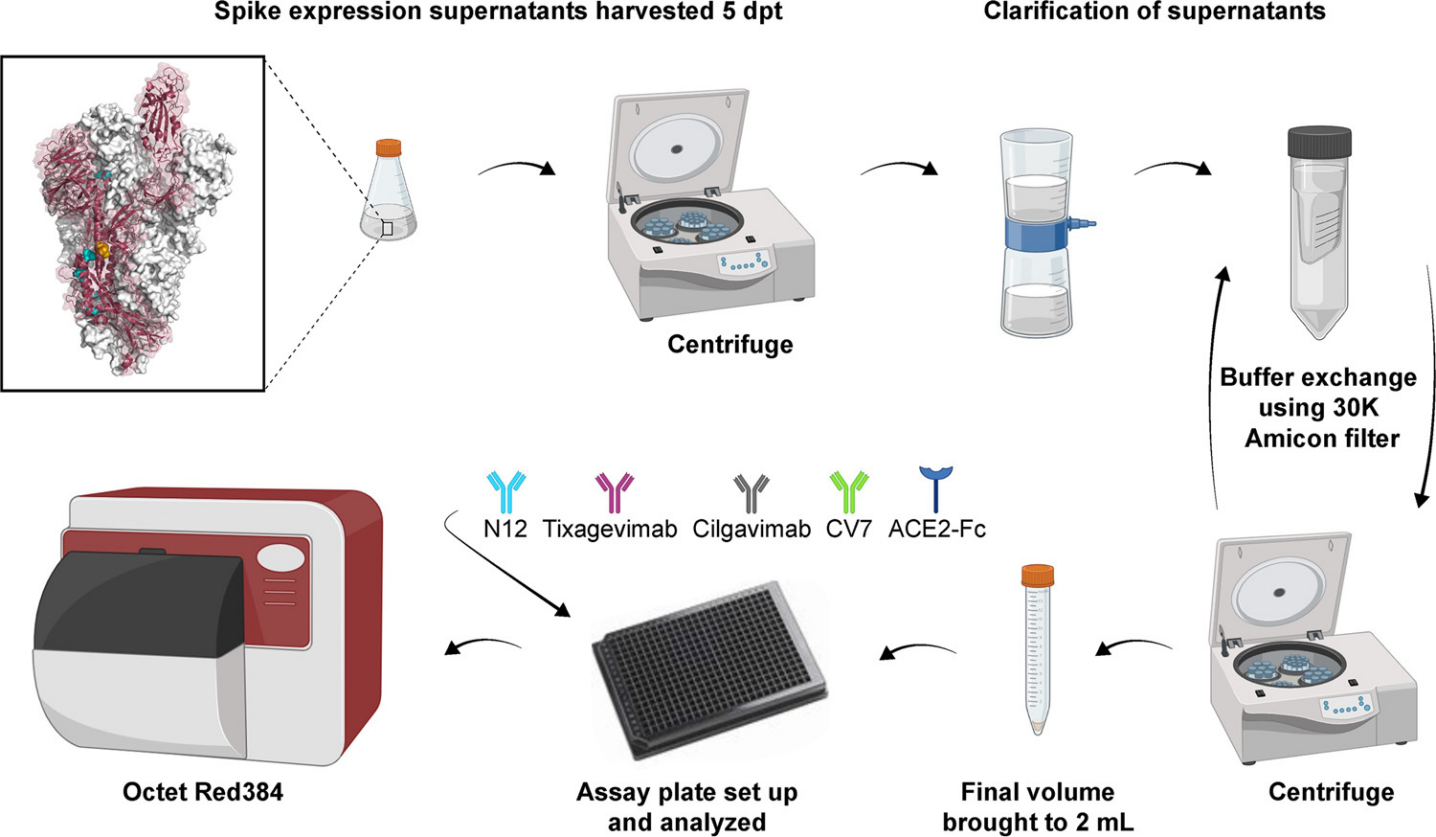
HexaPro spike trimer construct and spike trimer expression assay flow. Light blue shows proline substitutions in trimer construct, and gold shows GSAS site. ACE2, angiotensin-converting enzyme 2; dpt, days posttransfection. Figure created with BioRender.com.

The assay was first optimized to minimize the time from transfection to testing by eliminating a full protein purification step. Recombinant SARS-CoV-2 spike proteins were designed to include several features that would increase protein stability and expression yield to ensure sufficient quantity for testing straight from the expression supernatant (25, 26). Specifically, the current HexaPro design iteration included a further four proline substitutions and a GSAS substitution at the furin cleavage site, which disrupted cleavage between the S1/S2 subunits (Fig. 1). Additionally, for use in the BLI assay, a C-terminal streptavidin tag in the HexaPro construct allowed loading of the trimer onto high-precision streptavidin biosensor tips (SAX tips). An assay procedure flow diagram describing the evaluation of spike trimer expression is shown in Fig. 1. In order to function as an effective screening assay, the full-length trimer was required, to capture co-occurring substitutions that lie outside the RBD. A comparison of equilibrium dissociation constant (*K_D_*) values for different antigen:analyte formats is shown in Table SA1 in the supplemental material.

**FIG 2:**
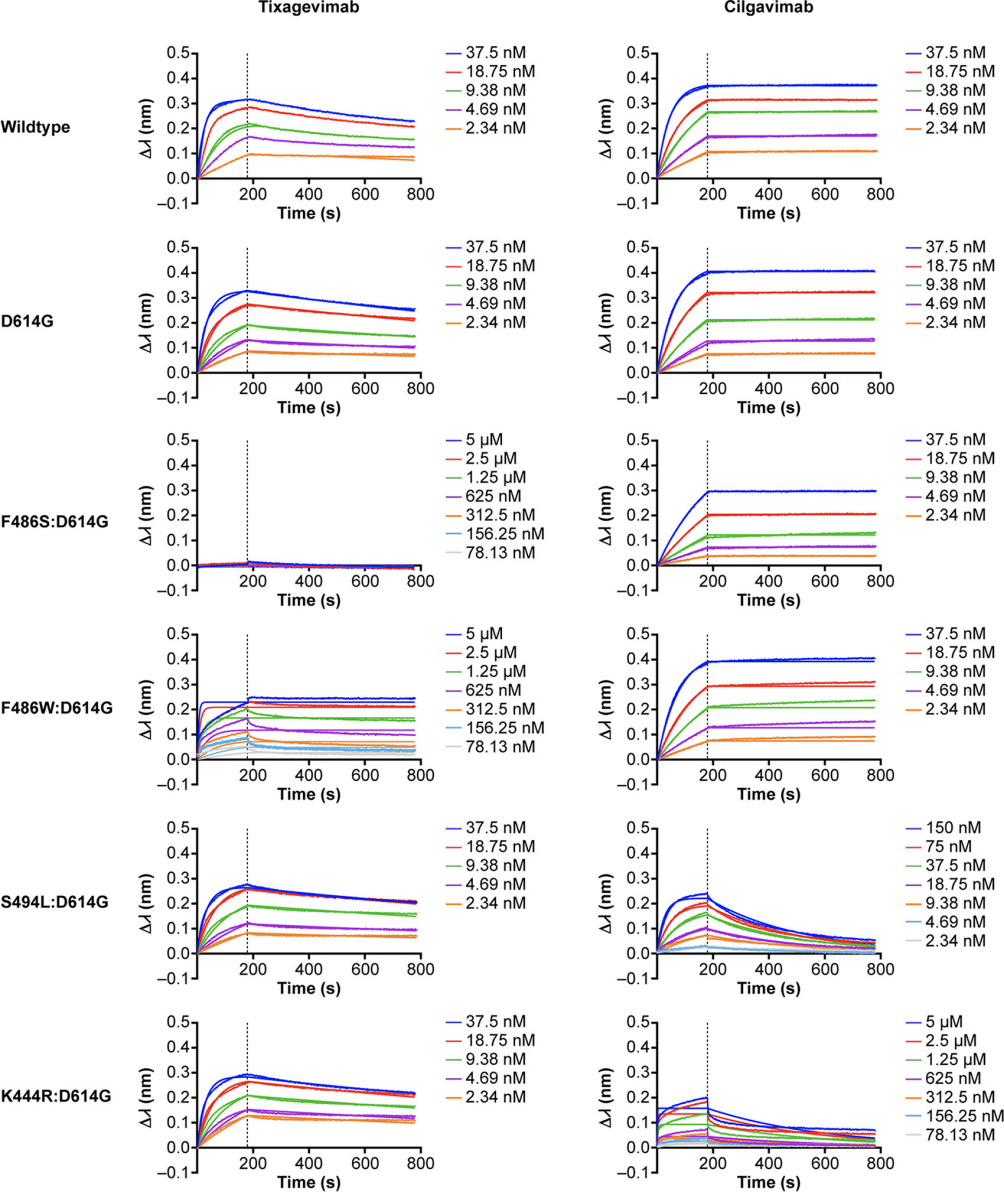
Binding traces for each spike protein in the qualification panel against the two test antibodies. Spike proteins were loaded on the biosensor tips. Indicated concentrations refer to either tixagevimab (left) or cilgavimab (right) in the association phase for each spike protein. The association phase was 180 s, followed by a 600-s dissociation phase. Binding is represented by wavelength shift (Δλ; measured in nanometers [nm]) as detected by the Octet instrument.

Six recombinant spike proteins were selected to assess the assay’s performance, including recombinant spike proteins containing substitutions within the tixagevimab or cilgavimab binding sites (27). The panel contained two reference proteins, D614G protein and wild-type HexaPro (D614). Four additional spike proteins were selected as containing substitutions that decrease affinity to tixagevimab (F486S:D614G and F486W:D614G) or cilgavimab (S494L:D614G and K444R:D614G) (Fig. 2). The panel selection ensured that the qualification results would be applicable to the different alterations of binding kinetics potentially encountered in postqualification screening.

### Acceptance/qualification.

Qualification acceptance was based on evaluation of assay precision (repeatability, set at a percent coefficient of variation [%CV] of ≤35%; interassay precision, %CV of ≤40%; and intermediate precision, %CV of ≤45%), limit of detection (LOD), limit of quantitation (LOQ; prespecified as ≤0.1084 nm), range, linearity, and specificity. Two series of experiments were performed: series 1 evaluated assay precision, LOD, LOQ, range, and linearity, and series 2 evaluated specificity.

**FIG 3:**
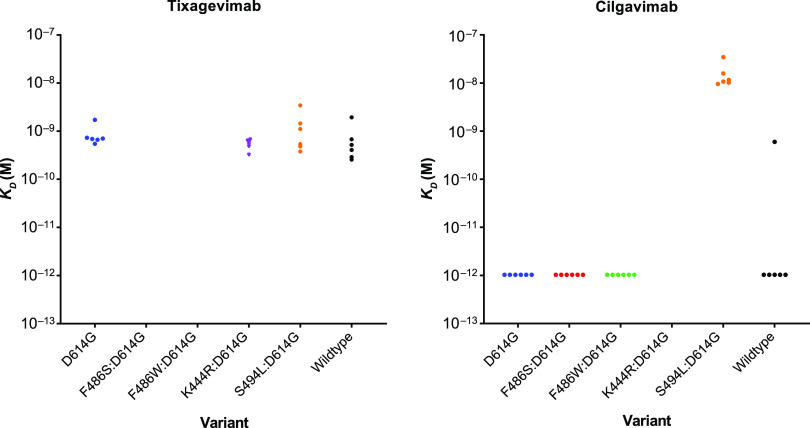
Precision represented by *K_D_* values against variant replicates for tixagevimab and cilgavimab. *K_D_* values of <1E−12 M were considered below the LOD. *K_D_*, equilibrium dissociation constant; LOD, limit of detection.

**(i) Precision.** The *K_D_* values were assessed for precision for tixagevimab or cilgavimab binding (Fig. 3). One outlier occurred with one replicate of the wild-type reference protein with cilgavimab, where the *K_D_* value was 6.69E−10 molar (M); this was designated an outlier on the basis that there was a nearly 1,000-fold difference in *K_D_* value compared with those of the other five replicates.

The repeatability %CV (within-assay precision) was estimated at 27%, the interassay precision was 34%, and the intermediate (overall) precision was 45%, each being within the prespecified acceptance criteria of ≤35%, ≤40%, and ≤45%, respectively (Table 1).

**TABLE 1:** Precision estimates for tixagevimab and cilgavimab, including and excluding the outlier

Parameter	Precision estimate [%CV (95% CI)]^*a*^	
	Including outlier	Excluding outlier
Repeatability	38 (22–145)	27 (0–59)
Interassay	105 (78–164)	34 (27–45)
Intermediate (overall)	122 (92–186)	45 (35–63)

a CV, coefficient of variation; CI, confidence interval.

The resulting *K_D_* values for each recombinant spike protein against either tixagevimab or cilgavimab are shown in Table 2. The *K_D_* values for binding to tixagevimab ranged between 4.91E−10 M and 8.55E−10 M. Most spike trimer proteins showed strong binding to cilgavimab (*K_D_* of <1E−12 M), except for the protein with S494L:D614G substitutions (*K_D_* of 1.59E−8 M). In routine testing, spike trimers with *K_D_* values of <1E−12 M against cilgavimab would be considered to have equivalent kinetics, meaning that the corresponding substitutions were unable to overcome the avidity effect and the MAb did not dissociate during the dissociation phase. Thus, *K_D_* values that registered as <1E−12 M in qualification were assigned a value of 1E−12 M for the purpose of determining fold changes and correlative comparison. *K_D_* values were not estimated for three sets of spike proteins: those with F486S:D614G (no tixagevimab binding), F486W:D614G (poor tixagevimab binding), and K444R:D614G (poor cilgavimab binding) substitutions.

**TABLE 2:** Mean *K_D_* value by variant

Variant	Mean *K_D_* value (M [95% CI]) for^*a*^:	
	Tixagevimab	Cilgavimab
D614G	7.57E−10 (5.4E−10, 1.1E−9)	<1E−12 (NE)
Wild-type	4.91E−10 (3.4E−10, 6.9E−10)	<1E−12 (NE)
S494L:D614G	8.55E−10 (6.0E−10, 1.2E−9)	1.59E−8 (1.1E−8, 2.2E−8)
K444R:D614G	5.37E−10 (3.8E−10, 7.5E−10)	NA (NE)
F486S:D614G	NB (NE)	<1E−12 (NE)
F486W:D614G	NA (NE)	<1E−12 (NE)

a *K_D_*, equilibrium dissociation constant; M, mol/L; CI, confidence interval; NE, 95% CI was not evaluable; NA, not available due to poor binding with inaccurate curve fitting; NB, no binding. 1E−12 M was used for *K_D_* values of <1E−12 M, and 95% CI was not calculated for variants with a mean *K_D_* of <1E−12 M.

Fold shift calculations for the *K_D_* values of tixagevimab and cilgavimab with each spike protein compared with those of the reference D614G spike protein are shown in Table 3.

**TABLE 3:** Tixagevimab and cilgavimab *K_D_* fold shift calculations with respect to the value for the reference D614G protein^*a*^

Variant	Mean *K_D_* fold shift calculation (95% CI) for^*b*^:	
	Tixagevimab	Cilgavimab
Wild-type	0.65 (0.26–1.60)	1 (NE)
S494L:D614G	1.12 (0.46–2.79)	15,855 (11,000–23,000)
K444R:D614G	0.71 (0.35–1.43)	NA
F486S:D614G	NB	1 (NE)
F486W:D614G	NA	1 (NE)

a M, mol/L; *K_D_*, equilibrium dissociation constant; CI, confidence interval; NE, 95% CI was not evaluable; NA, not available due to poor binding with inaccurate curve fitting; NB, no binding.

b 1E−12 M was used for *K_D_* values of <1E−12 M to calculate fold-change.

**(ii) LODs.** The LOD and LOQs (lower LOQ [LLOQ], lower LOD [LLOD], upper LOD [ULOD], and upper LOQ [ULOQ]) of binding with D614G protein were determined for five analytes (N12, tixagevimab, cilgavimab, CV7, and ACE2-Fc) at seven different concentrations. The plate layout is described in Table SA1. LODs were reported as response values measured in nanometers. The LLODs ranged between <0.0001 nm (ACE2-Fc) and 0.0241 nm (cilgavimab), and the ULODs ranged between 0.4175 nm (tixagevimab) and 1.8647 nm (CV7) (Table 4). The LLOQs were calculated using the 0 nM reference wells and were set at 0.1000 nm for all analytes, which was within the prespecified acceptance criterion of ≤0.1084 nm. For CV7, only the highest three concentrations (5 μM, 2.5 μM, and 1.25 μM) consistently showed binding responses above the LLOQ (0.1000 nm) and were thus used to calculate *K_D_* in all cases. The ULOQ was unique to each analyte, and the values ranged between 0.2652 nm (ACE2-Fc) and 1.3490 nm (CV7) (Table 4).

**TABLE 4:** Limits of detection and quantitation by analyte

Analyte^*a*^	Value (nm) for^*b*^:			
	LLOD	ULOD	LLOQ	ULOQ
N12	0.0135	0.4277	0.1000	0.3270
Tixagevimab	0.0006	0.4175	0.1000	0.3497
Cilgavimab	0.0241	0.4563	0.1000	0.3811
CV7	0.0044	1.8647	0.1000	1.3490
ACE2-Fc	<0.0001	0.4252	0.1000	0.2652

a ACE2, angiotensin-converting enzyme 2.

b LLOD, lower limit of detection; LLOQ, lower limit of quantitation; ULOD, upper limit of detection; ULOQ, upper limit of quantitation; nm, nanometer.

**FIG 4:**
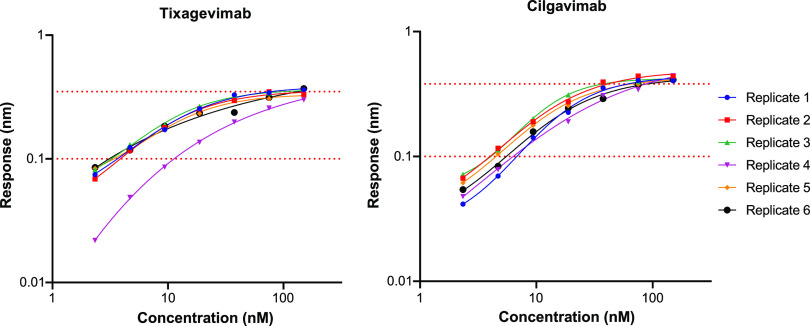
Range and linearity by analyte for tixagevimab and cilgavimab. Dotted lines show lower limit of quantitation and upper limit of quantitation. The ULOQs were 0.3497 nm for tixagevimab and 0.3811 nm for cilgavimab.

**(iii) Range and linearity.** The ranges of the BLI assay results for tixagevimab and cilgavimab are shown in Fig. 4, with the linear ranges depicted as the regions between the dotted red lines. All traces were included during qualification, to establish the linear ranges. Traces outside the linear ranges were excluded from the *K_D_* calculations when comparing with 50% inhibitory concentrations (IC_50_) for the correlation.

**FIG 5:**
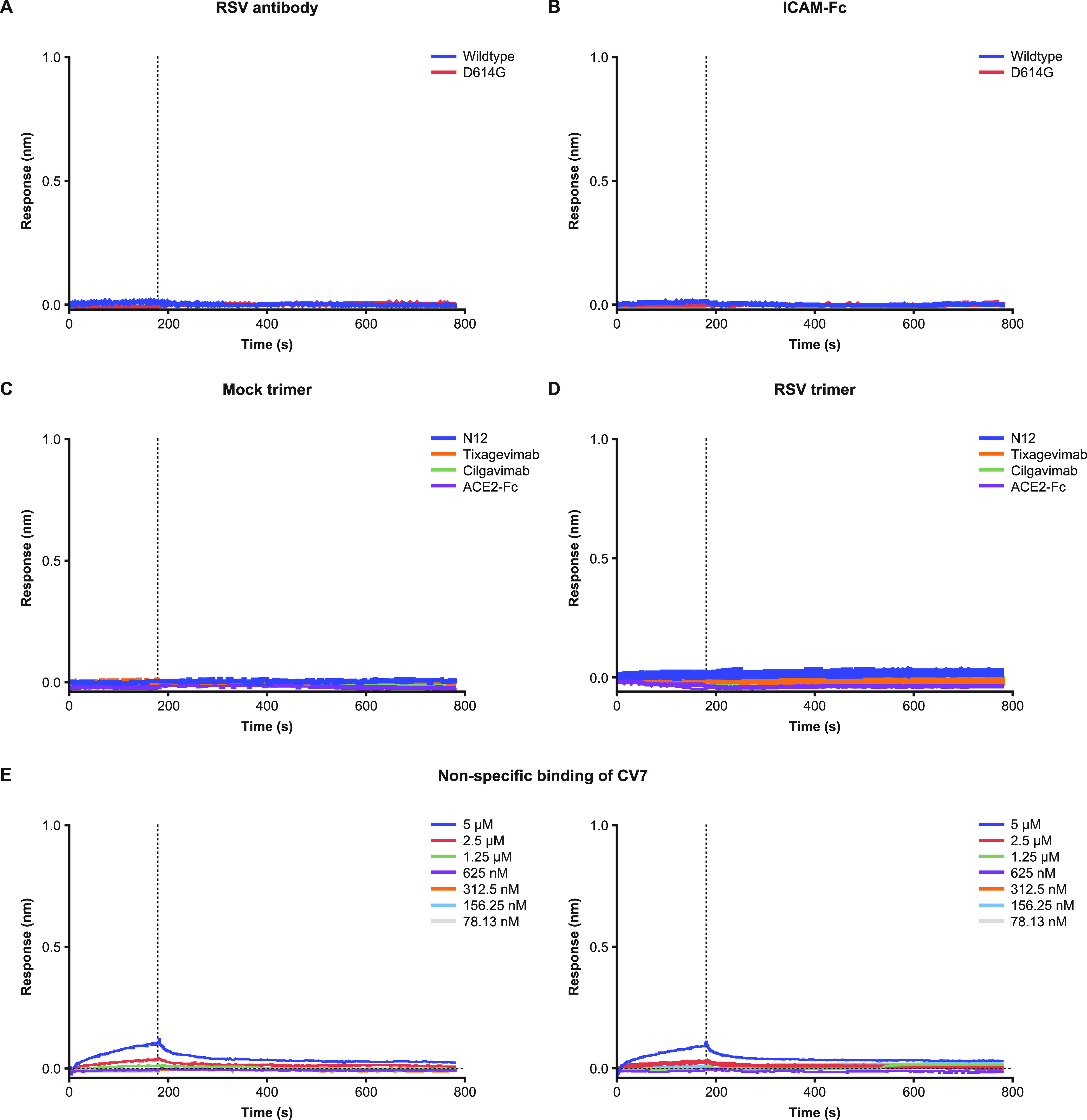
Assay specificity. (A) SARS-CoV-2 spike trimers versus non-SARS-CoV-2 anti-RSV 1g7-TM-YTE antibody. RSV antibody used in the association phase with either the wild-type trimer or D614G. (B) SARS-CoV-2 spike trimers versus ICAM-Fc. ICAM-Fc used in the association phase with either the wild-type trimer or D614G. ICAM-Fc started at 1 μM to match concentrations of ACE2-Fc. (C) Nonspecific binding with mock trimer. Mock transfection was used as trimer in the load phase of the assay. Association and dissociation are shown for the four analytes that did not show any evidence of binding. (D) Nonspecific binding with RSV A2 trimer. RSV trimer was used in the load phase of the assay. Association and dissociation are shown for the four analytes that did not show any evidence of binding. (E) CV7 versus mock trimer and RSV A2 trimer. Left, nonspecific binding of CV7 with mock trimer with the corresponding concentrations listed. Right, nonspecific binding of CV7 with RSV trimer with the corresponding concentrations listed. Mock trimer is buffer-exchanged transfection supernatant. Panels show the association (0 to 180 s) and dissociation (180 to 780 s) phase for each test. ICAM, intercellular adhesion molecule; RSV, respiratory syncytial virus; SARS-CoV-2, severe acute respiratory syndrome coronavirus 2.

**(iv) Specificity.** Four specificity tests were conducted to evaluate nonspecific binding of recombinant spike proteins. To evaluate the binding of the SARS-CoV-2 spike trimer with non-SARS-CoV-2 antibodies, the binding of either the wild-type reference or D614G trimer to a MAb specific to respiratory syncytial virus (RSV; MAb 1g7-TM-YTE) was assessed. RSV 1g7-TM-YTE contains the same TM-YTE modifications as tixagevimab and cilgavimab but recognizes the prefusion F protein of RSV. No evidence of 1g7-TM-YTE binding was observed in the association phase, and the maximum response (~0.005 nm) was below the LLOQ and within the background of the assay (Fig. 5A). It was not possible to calculate binding kinetics from the sensogram traces, and the results met the acceptance criteria. The binding of the wild-type reference and D614G spike trimers to intercellular adhesion molecule (ICAM)-Fc was also assessed in another measurement of specificity, mimicking the ACE2-Fc construct used in the assay. Here, no evidence of ICAM-Fc binding was observed in the association phase, and the maximum response (~0.015 nm) was below the LLOQ and within the background noise of the assay (Fig. 5B). Since the starting concentration for ICAM-Fc was the same as for ACE2-Fc (1 μM), the Fc tag appeared not to be involved in the binding of ACE2 to the spike trimer.

To evaluate any nonspecific binding in the loading phase, a mock buffer-exchanged transfection supernatant was used in place of a spike expression vector. Buffer was exchanged following the same procedure as for the trimer variants but utilizing a mock transfection protocol. The resulting kinetics calculations showed no evidence of nonspecific binding interference (Fig. 5C). The sensogram traces did not reach 0.8 nm in the loading phase, remained well below the LLOD, and met the acceptance criteria (Fig. SA1).

To evaluate possible binding of analytes to biosensor tips, an RSV A2 trimer construct was selected, as it contained a streptavidin tag compatible with the SAX tips used in the assay (Fig. SA1). RSV trimer binding was assessed for five analytes (Table SA2), with 1g7-TM-YTE included as a control antibody. For all analytes except CV7, no traces were detected in the association phase (Fig. 5D), suggesting that, based on kinetic calculations, tixagevimab and cilgavimab were free of nonspecific binding interference.

Minimal traces were detected for CV7 at 5 μM and 2.5 μM against both the mock trimer and the RSV antigen (Fig. 5E). As a result, the double reference subtraction method was used with CV7 in routine testing to remove the contribution of nonspecific binding at higher concentrations.

### Comparison of BLI and pseudotype SARS-CoV-2 spike variant neutralization susceptibility assays.

**FIG 6:**
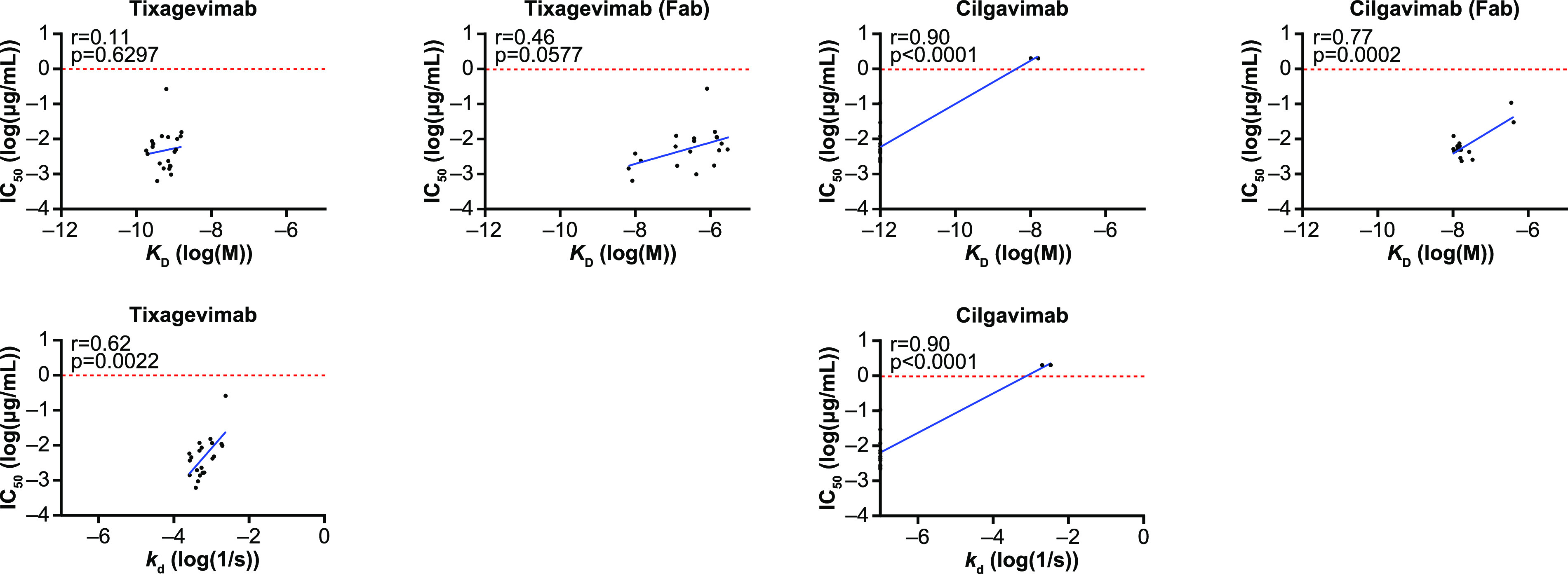
Correlation between pseudotyped-virus neutralization assay and BLI assay for tixagevimab and cilgavimab and for tixagevimab Fab and cilgavimab Fab. BLI, biolayer interferometry; Fab, antibody fragment; IC_50_, 50% inhibitory concentration; *K_D_*, equilibrium dissociation constant; *k_d_*, dissociation constant.

The correlations between MAb potencies determined by a lentivirus-based SARS-CoV-2 spike pseudovirus neutralization susceptibility assay and kinetic data determined by the BLI assay are shown in Fig. 6. Tixagevimab and cilgavimab potencies (IC_50_ values) were plotted against the corresponding *K_D_* values for the evaluated spike trimers. The IC_50_ and *K_D_* values showed minimal correlation, with a weak Pearson’s coefficient calculable for tixagevimab. Despite the strong correlation observed with cilgavimab, the result appeared to be skewed by unmeasurably low *K_D_* values for the majority of the evaluated spike trimers. As full immunoglobulins (IgGs) were used in the determination of binding kinetics, it is likely that antibody avidity may have acted as a potential confounder, resulting in the clustering of data points based on the measured *K_D_* values. Due to this possibility, antibody fragments (Fabs) of tixagevimab and cilgavimab were constructed and analyzed using the same BLI assay method (adjusted starting concentration of 300 nM). The use of 300 nM Fab achieved a maximum response of ~0.05 nm against trimers, where a shift was detected but was insufficient for calculating kinetics. Increasing the starting concentration to 2.5 μM resulted in restoration of the maximum responses back to 0.1 to 0.15 nm for this subset of spike proteins. The binding kinetics with monovalent Fabs showed higher correlations of IC_50_ and *K_D_* values (*r* = 0.46 and *r* = 0.77 for tixagevimab Fab and cilgavimab Fab, respectively). To further investigate the effects of antibody avidity, we also focused on the dissociation constant (*k_d_*), which in theory is an average measure of the IgGs that are fully disassociating from the antigens and accounts for the rapid disassociation and reassociation due to their bivalent nature. This inverse relationship with complex stability thus represents avidity. The correlation between the *k_d_* and IC_50_ values of cilgavimab again appeared to be skewed due to unmeasurably low *k_d_* values. However, the *k_d_* value of tixagevimab showed a correlation with the IC_50_ value (*r* = 0.62) that was similar to the correlation for tixagevimab Fab. Taken together, these results reflect a better comparison of the antibody binding kinetics and their neutralization potencies.

## DISCUSSION

In the ongoing COVID-19 pandemic, due to the emergence of SARS-CoV-2 variants, the need for new therapies to protect those individuals who remain at risk of COVID-19 persists. These include unvaccinated individuals, individuals who are unable to mount an adequate immune response following vaccination, and individuals with breakthrough infections despite full vaccination. SARS-CoV-2-neutralizing MAbs represent an approach for both prevention and treatment of COVID-19 for individuals who may not optimally respond to or are ineligible to receive a COVID-19 vaccine. Regulatory guidance supports the use of MAbs in combination as a potential strategy to avoid neutralization escape compared with the risk of escape with individual MAbs (28). To support the regulatory requirements of assessing potential changes in the susceptibility of emerging variants to AZD7442, a high-throughput BLI assay was developed for phenotypic screening of potential reduced MAb affinity against SARS-CoV-2 spike proteins containing sequence changes identified in circulating variants. Specific advantages of this technique include higher throughput, potential to achieve lower coefficients of variation than with cell-based assays like neutralization assays, and bypassing the need for protein purification. This approach was anticipated to allow rapid assessment of binding affinity for SARS-CoV-2 spike proteins, potentially obviating or reducing the need to perform the more technically demanding live-virus and/or pseudovirus neutralization assays.

The interrun, intrarun (repeatability), and intermediate (total) precision estimates with this assay were all within the prespecified acceptance criteria (following the exclusion of an outlier), with an LLOQ of 0.1 nm across all analytes and spike variants. Moreover, the resulting *K_D_* of each recombinant spike protein offered an opportunity to quantify the impacts of amino acid substitutions on MAb binding affinity compared with that of the D614G reference trimer. Here, tixagevimab’s binding affinity to spike proteins containing F486S:D614G substitutions was significantly reduced, while cilgavimab’s binding affinity was maintained. Both tixagevimab and cilgavimab together nevertheless retained neutralizing potency against a pseudovirus SARS-CoV-2 spike variant harboring the F486S:D614G substitutions. The BLI assay further showed a high degree of specificity. The specificity investigations determined that spike proteins were not bound by MAbs targeting other epitopes (RSV MAb 1g7-Tm-YTE) or the Fc tag. Importantly, alternative trimers (RSV F and mock) were not bound by tixagevimab and cilgavimab in the BLI assay. Collectively, the precision and selectivity estimates validated the BLI assay as fit for purpose for the objective of calculating and reporting fold shifts in binding affinity for each variant compared with the binding affinity for the reference.

Despite robust precision and specificity measurements, examination of the correlation between SARS-CoV-2 pseudotype virus neutralization and the SARS-CoV-2 spike protein BLI assay showed that the utility of the BLI assay in acting as a predictive measurement for *in vitro* neutralizing potency might be constrained by avidity effects from bivalent analytes. Since the viral spike has three potential MAb binding sites (one on each of the three RBDs) and IgG is bivalent, a spike-IgG binding assay will by definition measure the avidity and not the affinity. To act as a useful screening assay to assess the interaction of MAbs like tixagevimab and cilgavimab, assessments of binding affinity must be tightly correlated with MAb potencies. Unexpectedly, for multiple spike proteins, the binding of full-length IgGs as determined by the BLI assay did not correlate well with the results of the neutralization assay method. This contrasted with the correlations seen for antigen-binding Fabs, suggesting that avidity was a confounder. These data are consistent with reports from other laboratories showing that avidity strongly influences the off-rate of binding to SARS-CoV-2 or influenza envelope glycoprotein trimers (29, 30). Antibody avidity, an important characteristic of the normal humoral immune response after both infection and vaccination that typically increases (matures) over time, is suggested as a diagnostic tool to estimate the time of acquisition of infection (31, 32). However, the inherent variability in MAb-spike protein avidity may have contributed to the poor correlation between binding and MAb potencies. It is also possible that full-length IgGs bound with such high affinity and avidity compared with the Fabs that small changes were not detectable. This is unlikely to be a SARS-CoV-2-specific phenomenon, as a study of an anti-RSV MAb (palivizumab) has previously suggested that antibody binding valence plays a critical role in mediating viral neutralization through a mechanism that is likely unrelated to antibody size or binding avidity (33).

Antibody characterization by BLI has previously been described for SARS-CoV-2 and has aided in understanding the influence of antibody isotypes or monomeric versus polymeric forms of antibodies and structure/function studies (34–36). Additionally, it has been utilized for the characterization of antigenicity, as well as having utility in describing mechanisms of immune evasion by antibodies (37, 38). Despite qualification and strong assay performance characteristics, the BLI assay results did not demonstrate high correlation with neutralizing antibody potencies, limiting its utility as a screening modality for clinical resistance and surveillance virology of MAbs like tixagevimab and cilgavimab. These results suggest that caution is needed when interpreting binding affinity as a predictor for neutralization. While optimizing assay development based on Fabs may remain a promising alternative approach, given their stronger correlations with neutralization potencies, the use of Fabs rather than clinical full-length antibodies as a predictor of neutralization potency may face regulatory challenges toward supporting clinical programs.

The limitations of this analysis include the use of a single, dedicated instrument (Octet Red384; Sartorius Corporation, Bohemia, NY, USA) for BLI assessments, which did not allow complete assessment of reproducibility. Also, as accepted reference values for tixagevimab and cilgavimab have not been established, accuracy could not be assessed. An additional limitation is the use of an imputed LOD for the assessment of precision, potentially biasing toward a lower estimate of precision. While it is not currently possible to determine kinetics for spike protein variants with severely reduced binding, future studies identifying recombinant proteins with kinetics similar to those of proteins with the spike substitutions F486W and K444R will help to build a larger panel for use in optimizing the assay when applied at relatively high concentrations.

Here, we described the development and qualification of a BLI assay for screening SARS-CoV-2 spike trimer variants against anti-SARS-CoV-2 MAbs. We showed that despite the measurements being precise and specific, the use of the clinical full-length MAbs results in a clear avidity effect that limits correlation with the neutralization assay. While this provides useful kinetic data as to how the component MAbs of AZD7442 interact with spike proteins, it does compromise the potential utility of the assay as an effective screening tool. To improve this strategy, the findings show that optimization of the assay with Fabs would overcome the avidity limitation and may warrant further exploration in future resistance monitoring programs.

## MATERIALS AND METHODS

The study plan followed the assay qualification guidelines set forth by the International Conference on Harmonisation in document Q2(R1) (27).

### Spike constructs.

Variant trimeric spike proteins were designed in a HexaPro spike construct to increase the expression yield. A previous iteration of the prefusion-stabilized spike construct used two proline substitutions (referred to as S-2P) capping a central helix adjacent to the heptad repeat one to prevent conformational change (26). However, HexaPro contained an additional four proline substitutions in combination with the two S-2P substitutions that were found to further enhance stability and significantly increase expression (25). HexaPro also contained a GSAS substitution at the furin cleavage site, which disrupted cleavage between the S1 and S2 subunits (Fig. 1). Collectively, these features increased the stability of the prefusion conformation of the protein, preventing conformational rearrangement to the more stable postfusion configuration (25).

For the BLI assay, a C-terminal streptavidin tag in the HexaPro construct allowed loading of the trimer onto high-precision SAX tips. Spike variants were cloned into a backbone construct containing these features. Variants with substitutions that overlapped a proline or the GSAS site were not tested using BLI but evaluated only by the pseudotyped SARS-CoV-2 spike variant neutralization susceptibility assay.

### Spike trimer expression.

To generate recombinant spike trimer proteins, sequence-confirmed spike trimer plasmids were transfected into HEK293x cells using 293fectin reagent. Five days following transfection, the suspension was centrifuged, and the supernatant passed through a 0.2-μm filter. The supernatant (20 mL) was equally split between two 30 kDa Amicon filter tubes and centrifuged again until <1 mL of supernatant remained above the filter. Phosphate-buffered saline (PBS; 10 mL) was added to each tube, and centrifugation was repeated until <1 mL of supernatant remained above the filter. The expression supernatant sample was brought up to a final volume of 2 mL in PBS, stored at 2 to 8°C, and tested within 2 days following PBS buffer exchange. Immediately prior to testing, the expression supernatant was diluted 10-fold in assay buffer (HBS-EP+ buffer; Cytiva, Marlborough, MA, USA) (Fig. 1).

### BLI assay overview.

In a BLI assay, the primary molecule is loaded onto the sensor tip, which results in an interference pattern detected by an Octet instrument and is measured as an increased wavelength shift in nm. After the antigen is immobilized, the tips are transferred into solutions of various concentrations of analytes for the association phase. As the analyte binds, the interference pattern changes, and the wavelength shift increases further. The sensor tips are then moved into buffer for the dissociation phase, in which a decrease in the measured wavelength shift is observed as the analyte dissociates.

The BLI assay was used to evaluate the binding kinetics of tixagevimab and cilgavimab against spike trimer proteins. All assays were run on the same Octet Red384 instrument. Following a 60-s equilibration step with SAX tips in HBS-EP+ buffer, spike trimer was loaded until the corresponding wavelength shift reached 0.8 nm. This step enabled the concentration to be normalized across proteins in the absence of full purification and characterization. The tips were then moved into wells in the following sequence: (i) a second baseline step with fresh assay buffer for 2 min, (ii) analyte for a 3-min association step, and (iii) assay buffer for a 10-min dissociation step. The dissociation and second baseline steps used the same wells to take advantage of the interstep correction in the analysis phase.

Six recombinant spike proteins were selected to assess assay performance (Table 2). Binding curves from the association and dissociation steps were fitted using a 1:1 binding model to determine *K_D_* values; these were compared with the values for the two reference proteins (wild-type HexaPro [D614] and D614G protein), and the fold shifts relative to the *K_D_* values for trimer proteins containing the D614G substitution were determined. Proteins containing F486W:D614G exhibited insufficient curve fitting and were excluded from precision calculation. Binding profiles for F486S:D614G and F486W:D614G spike proteins were consistent across the different concentrations evaluated but without reportable *K_D_* values for precision estimates. The fold shift change for S494L:D614G against cilgavimab was significant. However, the mean *K_D_* values for F486W:D614G against tixagevimab and K444R:D614G against cilgavimab were unreliable and were excluded (*R*^2^ < 0.95 across all replicates (39); this was evident as a mismatch between fitted curves and measured profiles (Fig. 2)). The mismatch between on and off rates for the fitted curves versus measured traces meant that *K_D_* was not reflective of the true kinetic profile.

These data were compared with the pseudotyped SARS-CoV-2 neutralization susceptibility assay data to establish a correlation between MAb binding affinities and neutralization potencies. The curve equations were used to calculate the *K_D_*, *k_d_*, and association constant (*k_a_*).

**(i) Plate layout.** A typical plate included five analytes: tixagevimab, cilgavimab, N12, CV7, and an ACE2 construct containing an Fc tag (ACE2-Fc). N12 and CV7 were positive-control antibodies to confirm correct folding of the trimer protein. N12 and CV7 bind to unique regions on the spike trimer and do not compete with each other or with tixagevimab or cilgavimab. ACE2-Fc provided an additional control to further verify proper spike trimer folding by demonstrating antigen-receptor binding. For the five analytes, each plate row represented a different concentration, with the highest concentration in row A and following a 2-fold dilution scheme down to row M. Row O was the reference and contained only assay buffer.

**(ii) Qualification acceptance criteria and evaluation.** Qualification acceptance was based on evaluation of precision (repeatability, interassay, and intermediate), LOD, LOQ, range, linearity, and specificity. A single designated Octet Red384 instrument was used for this assay, and therefore, reproducibility was not evaluated. No predefined acceptance criteria were set for LOD, range, or linearity.

Two series of experiments were performed to evaluate assay performance. Series 1 evaluated assay precision, LOD, LOQ, range, and linearity and used variants in which substitutions would have minimal impact on binding affinity or in which changes caused a decrease in binding affinity against either tixagevimab or cilgavimab. Series 2 evaluated specificity, i.e., binding in the presence of noninteracting analytes across four scenarios.

**(iii) Precision.** Precision was estimated in terms of repeatability (intrarun precision), interrun precision, and intermediate precision. Each operator processed each variant in triplicate, and the replicate data were used to evaluate repeatability and intermediate precision. The precision of the assay across all spike proteins (excluding the controls) was obtained using a linear mixed-effects model, and Bayesian statistical methods were used to estimate the mean values and the components of variability, reported as the median of the respective posterior distributions (see the supplemental material).

**(iv) LOD, range, and linearity.** Analytes were each tested at seven concentrations, with a reference well included that contained only assay buffer to evaluate background noise (Table SA2). A four-parameter logistic curve (4PL) was fitted to each analyte on each plate (against reference D614G protein only). All the curves for the same analyte were assumed to share the same lower asymptote, upper asymptote, and standard deviation. LLOD was set to the lower asymptote of the 4PL curve, as response values below this have no biological meaning, while ULOD was set to the upper asymptote of the 4PL curve. The LLOQ was 10 times the standard deviation above the mean background noise where the standard deviation was that of the zero-concentration response data, and the ULOQ was 3 times the standard deviation below the ULOD (27, 40). These settings ensured a functional linear range that could accommodate a minimum of three response traces. The 4PL curve was fitted to the data according to the following equation:

$$\text{ln}(\text{response})=\text{Min}+\frac{\text{Max}-\text{Min}}{1+{\left(\frac{\text{concentration}}{{\text{IC}}_{50}}\right)}^{m}}$$

In this equation, Min (minimum) represents the lower asymptote, Max (maximum) represents the upper asymptote, and *m* is the slope parameter.

The 4PL curves were fitted via ordinary least-squares. The range of the BLI assay was defined as the window of response values between LLOD and ULOD, and linearity was the window of response values between ULOQ and LLOQ.

**(v) Plate pass/fail criteria.** During analysis, three controls were included (N12, CV7, and ACE2-Fc) to confirm whether any reduction in binding to tixagevimab or cilgavimab was related to the recombinant spike protein and not related to expression or the plate. Failure of ACE2-Fc and either N12 or CV7 to show evidence of binding required the plate to be discarded and the assay to be repeated. In addition, the association curve fit must have had an *R*^2^ of ≥0.95.

The manufacturer’s guidance recommends that at least three to five concentrations of analyte be used to calculate *K_D_*, *k_a_*, and *k_d_*. Because many plates could have failed based on this criterion, the minimum number of acceptable concentrations was adjusted to the lower end of the manufacturer’s recommendation. Specifically, a minimum of three concentrations were selected for each control and the two test MAbs as a reasonable requirement to fit between the LLOQ and ULOQ while maintaining a 2-fold dilution scheme. The use of four or more concentration levels reduced the number of plates in which either MAb provided sufficient response values to within the required range.

### Pseudovirus neutralization assay.

The performance of the pseudovirus neutralization assay was as described previously (6), and the antibody binding potencies determined by this method were correlated with those determined by the BLI assay. To generate spike protein pseudotyped lentivirus, suspension cultures of FreeStyle 293 or Expi293 cells (Thermo Fisher, Waltham, MA, USA) were seeded and transfected with a third-generation HIV-based lentiviral vector expressing luciferase, along with packaging plasmids encoding SARS-CoV-2 spike protein with a C-terminal 19-amino-acid deletion, Rev, and Gag-Pol. Following collection 48 h later, removal of cell debris by centrifugation, and passage of the supernatant through a 0.45-μm filter unit, the pseudovirus was pelleted by ultracentrifugation and resuspended in Opti-MEM to provide a 100-fold-concentrated stock. For the pseudovirus neutralization assay, serial dilutions of MAbs were prepared in a 384-well microtiter plate and preincubated with pseudovirus for 30 min at 37°C, to which 293 cells that stably express human ACE2 were added. The plate was returned to the 37°C incubator, and 48 h later, luciferase activity was measured on an EnVision 2105 multimode plate reader (Perkin Elmer, Waltham, MA, USA) using the Steady-Glo luciferase assay system (Promega, Madison, WI, USA) according to the manufacturer’s recommendations.

The percentage of inhibition was calculated relative to the results for the pseudovirus-only control, with mean IC_50_ values determined based on quadruplicate measures by nonlinear regression using Prism version 8.1.0 (GraphPad).

### Generation of Fabs.

Antigen-binding fragments were generated by digesting full-length tixagevimab and cilgavimab with immobilized papain (Thermo Fisher). Fabs were further purified using a HiTrap MabSelect SuRe column (Cytiva).

### Data availability.

Data associated with this study are available in the text or in the supplemental material. Crystal structures of tixagevimab (AZD8895) and cilgavimab (AZD1061) have been deposited in the Protein Data Bank (https://www.rcsb.org) under the accession numbers 7L7D (AZD8895 + RBD) and 7L7E AZD8895 and (AZD1061 + RBD).
