# Supplementary material for: Qualification of a Biolayer Interferometry Assay to Support AZD7442 Resistance Monitoring
Source: Microbiol Spectr. 2022 Aug 22;10(5):e01034-22. doi: 10.1128/spectrum.01034-22 (PMC9704045; doi:10.1128/spectrum.01034-22)
Supplement: Supplemental file 1 — Supplemental material. Download spectrum.01034-22-s0001.pdf, PDF file, 0.7 MB [file spectrum.01034-22-s0001.pdf]

Appendix

Table A1. Comparison of K_D for different antigen : analyte formats

		Tixagevimab		Cilgavimab	
		**IgG K_D (M)**	**Fab K_D (M)**	**IgG K_D (M)**	**Fab K_D (M)**
**Wildtype**	**Trimer**	2.88E-10	6.40E-09	<1.0E-12	1.20E-08
	**RBD[3,4]**		6.56E-09		1.34E-08
**B.1.1.7[1]**	**Trimer**	1.72E-10	2.31E-08	<1.0E-12	7.80E-09
	**RBD[3,4]**		8.66E-09		1.10E-08
**B.1.351[2]**	**Trimer**	1.50E-10	5.96E-08	<1.0E-12	9.67E-09
	**RBD[3,4]**		4.68E-08		1.22E-08

[1]Spike substitutions for B.1.1.7 in this assay were the following:

HV69-:Y144-:N501Y:A570D:D614G:P681H:T716I:S982A:D1118H.

[2]Spike substitutions for B.1.351 in this assay were the following:

D80A:D215G:LLA241-:K417N:E484K:N501Y:D614G:A701V.

[3]The RBD constructs (amino acid 333-526) contained a his-tag and were loaded on penta-his biosensor tips; all assay steps were as described in the methods.

[4]RBD was loaded on the tips to the same 0.8 nm shift threshold as trimer. All K_D values for both IgG mAbs were <1E-12M (blocked out). As RBD is much smaller, more RBD antigen was loaded on the tips. This suggests IgG was able to bind two adjacent RBD antigens simultaneously, creating a strong avidity effect that severely reduced dissociation time, rendering it below the instrument detection limit.

Fab, antibody fragment; IgG, immunoglobulin G; K_D, equilibrium dissociation constant; RBD, receptor binding domain.

Table A2. Analyte concentrations (nmol/L) for establishing assay parameters

Row	N12	tixagevimab	cilgavimab	CV7	ACE2-Fc
A	50	150	150	5000	1000
C	25	75	75	2500	500
E	12.5	37.5	37.5	1250	250
G	6.25	18.75	18.75	625	125
I	3.13	9.38	9.38	312.5	62.5
K	1.56	4.69	4.69	156.25	31.25
M	0.78	2.34	2.34	78.13	15.63
O	0	0	0	0	0

ACE2, angiotensin-converting enzyme 2.

Statistical evaluation

Precision across all spike variants by linear mixed-effects model

$$y_{ijkl} = Variant_i + mAb_j + A_k + e_{ijkl} \qquad (1)$$

Where y_{ijkl} is the $\ln(KD)$ value of the lth replicate for the i^{th}variant, the jth mAb ran on the kth assay;

$Variant_i$ is the mean effect of the ith virus;

mAb_j is the mean effect of the jth mAb

$A_k \sim N(0, \sigma_R^2)$ is the random effect of the kth assay, σ_R is the inter-assay variation; and

$e_{ijkl} \sim N(0, \sigma_e^2)$ is the residual error, σ_e is the within-assay variation (repeatability).

Intra-assay (repeatability), inter-assay, and intermediate precision were calculated using the following formulas:

$$\%CV_{intra} = 100\% \times \sqrt{e^{\sigma_e^2} - 1}$$

$$\%CV_{inter} = 100\% \times \sqrt{e^{\sigma_R^2} - 1}$$

$$\%CV_{intermediate} = 100\% \times \sqrt{e^{\sigma_e^2 + \sigma_R^2} - 1}$$

Bayesian statistical methods and prior distributions

Bayesian methods were used to estimate the means and the components of variability, reported as the median of the respective posterior distributions. The log shift of one variant relative to the reference variant was calculated as $\delta_v = \mu_v - \mu_0$,

where μ_0 is the mean of the reference variant, μ_v is the mean of the variant ν. Fold-shift was calculated as $\exp(\delta_v)$ and reported as the median of the posterior distribution. Bayesian methods required prior information to be blended with the study data, yielding the posterior distribution used in statistical analysis. For the prior distributions, normal distributions were provided for the means, and half-Cauchy distributions were provided for SD parameters (**Table A3**).

Table A3. Prior distributions

	**Prior distribution**
Mean effect	Normal distribution with mean taken from the estimates from fitting model (1) using REML-based method, SD 0.35
SD for variance components	Half-Cauchy with scale 0.4

REML, restricted (or residual, or reduced) maximum likelihood; SD, standard deviation.

Figure A1. Assay traces for mock trimer (left) and RSV trimer (right)

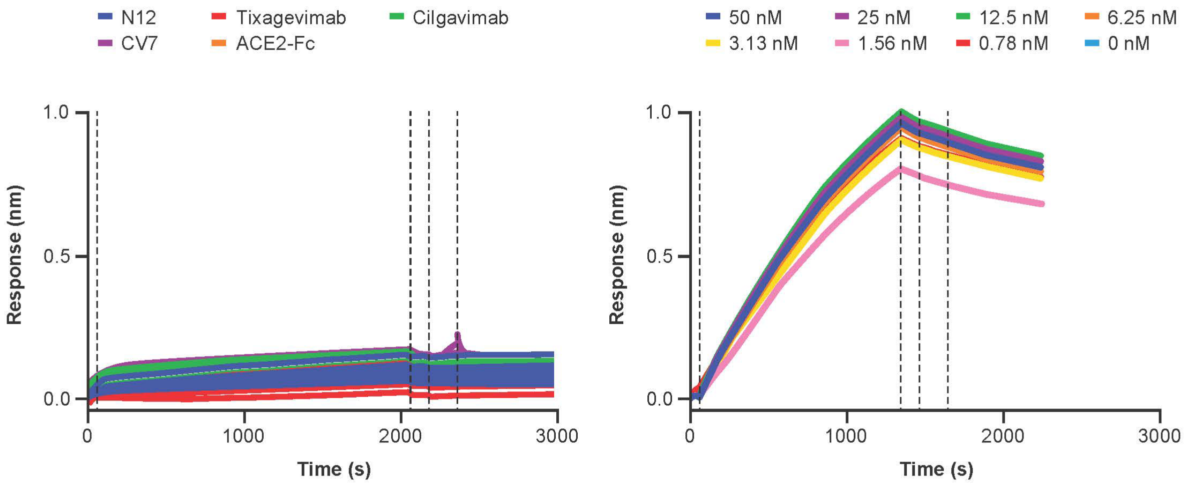

Left: Mock transfection used as trimer in the load phase. Baseline: 0-60s; load: 60-2060s; baseline: 2060-2180s; association: 2180-2360s; dissociation: 2360-2960s. Right: RSV F trimer added in load phase. Baseline: 0-60s; load: 60-1347s; baseline: 1347-1467s; association: 1467-1647s; dissociation: 1647-2247s. Representative traces show RSV trimer with N12 as the analyte at the designated concentrations.
